# The GBIF Integrated Publishing Toolkit: Facilitating the Efficient Publishing of Biodiversity Data on the Internet

**DOI:** 10.1371/journal.pone.0102623

**Published:** 2014-08-06

**Authors:** Tim Robertson, Markus Döring, Robert Guralnick, David Bloom, John Wieczorek, Kyle Braak, Javier Otegui, Laura Russell, Peter Desmet

**Affiliations:** 1 Global Biodiversity Information Facility, Copenhagen, Denmark; 2 University of Colorado, Boulder, Colorado, United States of America; 3 University of California, Berkeley, Berkeley, California, United States of America; 4 University of Kansas, Lawrence, Kansas, United States of America; 5 Research Institute for Nature and Forest (INBO), Brussels, Belgium; The New York Botanical Garden, United States of America

## Abstract

The planet is experiencing an ongoing global biodiversity crisis. Measuring the magnitude and rate of change more effectively requires access to organized, easily discoverable, and digitally-formatted biodiversity data, both legacy and new, from across the globe. Assembling this coherent digital representation of biodiversity requires the integration of data that have historically been analog, dispersed, and heterogeneous. The Integrated Publishing Toolkit (IPT) is a software package developed to support biodiversity dataset publication in a common format. The IPT’s two primary functions are to 1) encode existing species occurrence datasets and checklists, such as records from natural history collections or observations, in the Darwin Core standard to enhance interoperability of data, and 2) publish and archive data and metadata for broad use in a Darwin Core Archive, a set of files following a standard format. Here we discuss the key need for the IPT, how it has developed in response to community input, and how it continues to evolve to streamline and enhance the interoperability, discoverability, and mobilization of new data types beyond basic Darwin Core records. We close with a discussion how IPT has impacted the biodiversity research community, how it enhances data publishing in more traditional journal venues, along with new features implemented in the latest version of the IPT, and future plans for more enhancements.

## Introduction

Natural history collection records and data collected in citizen science efforts represent irreplaceable information about our biosphere. The value of these legacy data sources will increase as landscape and climate change accelerates and species-environment steady-state conditions decline [1]. In order for biocollections to be utilized to their full potential, there must be widespread access to the data they contain [2]–[3]. Many natural history collections, however, still struggle to mobilize data [4] and neither scientists nor the public have sufficient access to these resources.

Mobilizing biodiversity data en masse in ways that maximize open access and reuse require a robust and easily usable infrastructure. Wieczorek et al. [5] discuss the need for data to be made accessible, discoverable, and integrated, and further relate challenges to each of these endeavors. Integration can, in part, be achieved through the utilization of community-developed metadata standards such as Darwin Core [5]. Darwin Core is a vocabulary, or set of terms, that describe biodiversity data. These terms, comprising the Darwin Core standard (http://rs.tdwg.org/dwc/), have been vetted rigorously for utility by the biodiversity research community and are maintained through a well-defined governance process (http://www.tdwg.org/about-tdwg/process/).

A community standard helps to set the stage for interoperability and enhanced data discovery, but it is only one step in the larger process of data mobilization. Equally challenging is the development of tools that convert local data resources into published record sets that conform to those key community standards. The development of these publishing systems requires the recognition of a series of socio-technical challenges, including the generation of community buy-in and capacity building, and overcoming issues of scalability and sustainability as data sharing networks continue to grow.

In this paper, we describe a tool essential to the publication of biodiversity data: the Global Biodiversity Information Facility (GBIF) Integrated Publishing Toolkit (IPT, http://www.gbif.org/ipt/), a Java-based software package that provides the biodiversity community with a simple means to perform many necessary functions to publish biodiversity datasets on the web. The IPT is built upon lessons learned from previous data publishing methods, such as Distributed Generic Information Retrieval (DiGIR, http://digir.sourceforge.net/), the Biological Collection access Service for Europe (BioCASE, http://biocase.org/products/protocols/), and the Taxonomic Databases Working Group (TDWG) Access Protocol for Information Retrieval (TAPIR, http://www.tdwg.org/dav/subgroups/tapir/1.0/docs/tdwg_tapir_specification_2010-05-05.htm). We define the IPT, discuss the factors that led to its development and growth, and explain how it is being used and maintained. We also discuss near-term and longer-range community needs that can be met in future IPT releases.

## Methods

### The need for an Integrated Publishing Toolkit

There are estimated to be greater than two billion specimens in natural history collections worldwide [6], spanning thousands of collections and institutions. Although lacking physical vouchers, field-based observational datasets generated by citizen science efforts, such as eBird or iNaturalist, are quickly growing in size and scope, facilitated by the rapid adoption of mobile devices [7]. Harder to quantify are datasets about species and their distributions generated by laboratories worldwide. These data sources are often referred to as dark data [8] because they are largely ignored in data curation efforts, and thus are particularly prone to loss. In the biodiversity domain almost all non-published data are dark in this sense.

The key solution to the challenge of enhancing discovery and reuse of biodiversity data mirrors solutions in other domains, such as molecular biology, where deposition and publishing of sequences and genomic data to repositories such as GenBank became both a social norm and a requirement [9]. Proper social and technical approaches can convert the dark data coming from individual museums, citizen science projects, and laboratories to the integrated data of big science, allowing new fundamental questions to be asked from aggregates that could be not be addressed from any one individual data source [10].

Prototype development of biodiversity data-sharing networks at the turn of the millennium, such as the vertebrate biodiversity sharing networks [11]–[13], Ocean Biogeographic Information System (OBIS) [14], and GBIF [15], not only proved technological feasibility but showed that the demand was strong among users. For example, the vertebrate biodiversity sharing networks (*e.g.*, Mammal Networked Integrated System or MaNIS - http://manisnet.org/, Ornithological Research Networked Information System or ORNIS - http://www.ornisnet.org/, HerpNET - http://www.herpnet.org/, and FishNet II - http://www.fishnet2.net/, now collectively consolidated into VertNet - http://www.vertnet.org/) served over 2 billion records between June 2012 and June 2013 to users hungry for biodiversity data. More than 650 published works citing data from the VertNet networks have been published over the last decade.

**Figure 1:**
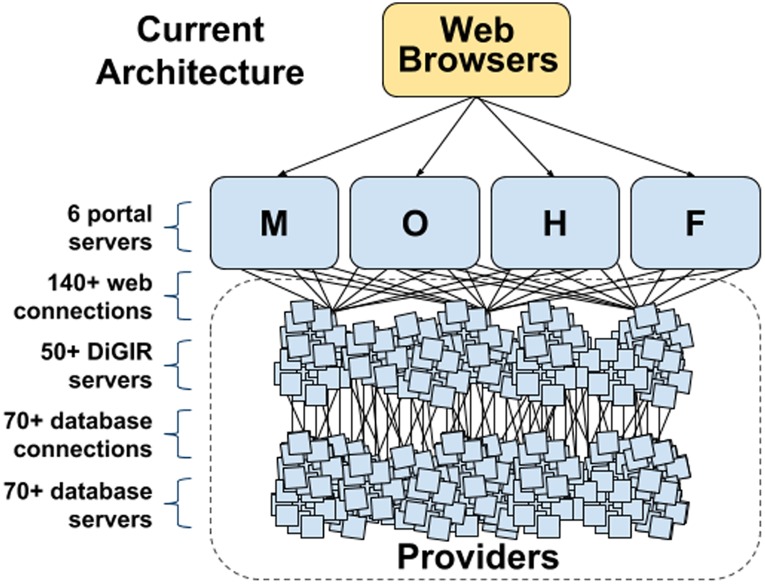
Design of the original vertebrate biodiversity networks, some of which are still active today, used a three-tiered system in which portals are connected to a layer of servers using the DiGIR protocol. This architecture requires hundreds of individual servers and hundreds more connections between them and the portal. The result is a network in which each element is a potential point of failure. The six portal servers consisted of the four shown and two additional mirror portals for the Mammal Networked Information System (MaNIS). Key: M = MaNIS, O = ORNIS, H = HerpNET, and F = FishNet II.

Biodiversity publishing systems were initially set up to be fully distributed, with aggregators serving as central nodes helping to facilitate data access, as opposed to serving the data directly themselves. Distributed networks rely on each data publisher to install and maintain the DiGIR, BioCASE or TAPIR middleware on a local server that allows connections to the host database, thereby allowing communication between the database and the aggregator, and in turn, the larger network. With these servers in place, datasets can be queried and results aggregated on-the-fly at a central portal and returned to the user. **Figure 1** shows this distributed network architecture for the vertebrate biodiversity networks.

Many distributed networks rely upon DiGIR, BioCASE or TAPIR middleware requests for data in the Access to Biological Collection Data (ABCD) and pre-standard Darwin Core formats (http://www.tdwg.org/standards/115/). The results are returned to a portal in an XML format. These middleware packages are installed on each and every machine across the distributed network and are complicated enough that most installations are beyond the technical capacity of the staff maintaining the servers. This has led to problems of sustainability and maintenance that can be overcome only through network management by specialized, and usually remote, support experts. To make clear the magnitude of the problem, many of the DiGIR providers on the GBIF network (111 of 161 when checked in June 2013) are no longer responding within a one minute timeout period when queried, and are likely not functioning.

For a small network, this complicated, distributed messaging system works, if imperfectly, but as the number of data publishers grows within a network, so do the number of connections, the number of queries to individual local databases, and the number of sources to be aggregated on-the-fly by the central node. Eventually, speed and efficiency begin to deteriorate, increasing both user frustration and the cost of network maintenance.

In an attempt to address these issues, the Global Biodiversity Information Facility (GBIF) began to harvest datasets from distributed data sources to a local server and to index them to improve response times. Although response times for queries to the network were faster, this new approach still had challenges: 1) it could take weeks to gather and index all the data provided by publishers; 2) formatting datasets for the index was inefficient, leading to high demand on the infrastructure; 3) data at the sources often changed between harvest and indexing, making it difficult to know if the data had been indexed completely, and; 4) the transfer protocols did not support the detection of record deletions. The way to verify that a record had been deleted was to check differences between indexing runs on different dates, which was an ever more daunting task as the volume of records grew.

An equally vexing problem with the networks continues to be data quality. The data published to aggregators remain “noisy”, and requires cleaning to be truly useful for biodiversity analysis [16]–[18]. One example of noisy data in all data provisioned by GBIF, as of March 2013, is that there are 321 distinct values used in the “country” field for records for which the country code is “US” (*e.g.*, the top five in order are USA, United State, U.S.A., United States of America, and US.). This problem is not unique to administrative units or controlled vocabularies. Many of the types of data described in Darwin Core, such as taxonomy, geospatial information sampling methods, and preparations face the same issues.

The previous network systems were not built to provide a constellation of support tools that could access these data, check for quality, and flag or cleanse the incorrect or questionable information. The full workflow of quality assessment and data cleaning was, and still is, a challenging task [19]. Making data harvesting from publishers to aggregators more efficient could pave the way to more centralized data cleaning tools that could help with fitness for use assessments across the network.

### Developing an Integrated Publishing Toolkit

The GBIF Integrated Publishing Toolkit was born from rethinking how data publication should work in the biocollections domain. The tool was based, in part, on the need for a simple, general publishing solution that was platform independent, could be easily managed by institutions, and leveraged existing metadata tools and standards, such as Ecological Markup Language (EML) [20]. The solution was a simple web-based publishing toolkit deployed as a Java application.

The initial development of the IPT happened concurrently with a related effort to reshape how records conforming to the Simple Darwin Core could be stored and shared by publishers in a common file format. This format, the Darwin Core Archive, was developed to provide a very simple mechanism to package and share data files. The specification for the structure of a Darwin Core Archive is given in the Darwin Core Text Guide (http://rs.tdwg.org/dwc/terms/guides/text/) [21]. Darwin Core Archives consist of one or more delimited text files of data, an XML file to describe the structure of and relationships between the data files, and a complementary metadata file to describe the dataset contained in the archive, using Dublin Core or the richer Ecological Markup Language [5]. Darwin Core Archives can be seen as a Research Object [22] with all of the associated ramifications for reproducible research, linking data, and the publication process.

**Figure 2:**
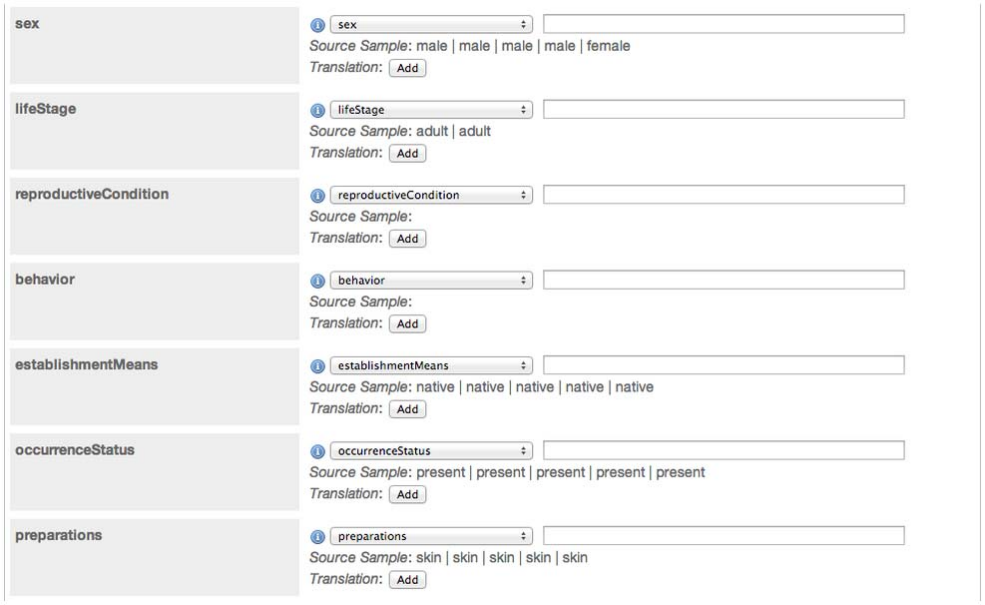
This screenshot of IPT shows how users map their local field headings to Darwin Core terms, an essential task for data publishers. The Darwin Core term names are on the left and terms loaded from a database or spreadsheet on the right, which are selected using dropdown menus. Fields that have the same name string in both Darwin Core and the publisher dataset are matched automatically, while those that do not match must be selected manually (via adrop-down list) by the “data publisher”.

**Figure 3:**
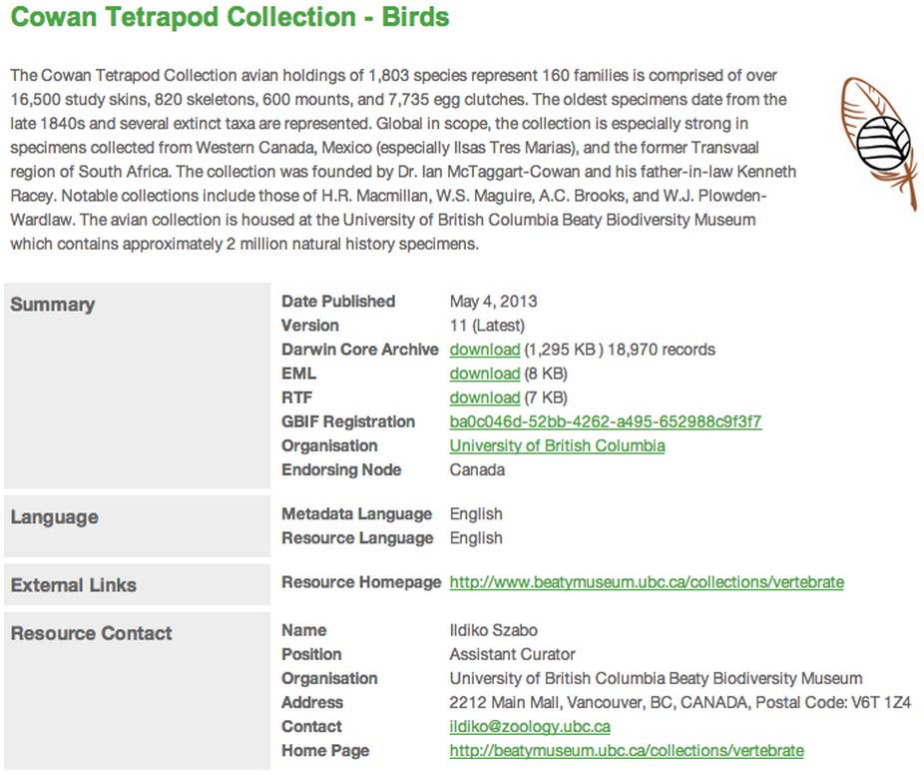
Example of an IPT summary page displaying some of the metadata provided for the dataset hosted by VertNet for the Cowan Tetrapod Collection of birds (http://ipt.vertnet.org:8080/ipt/resource.do?r=ubc_bbm_ctc_birds **).**

The guiding design principles of the IPT were to support how data publishers actually use their own databases, and to facilitate the public sharing of datasets with the fewest possible obstacles. As a result of these goals, the IPT was built to support simple publisher workflows, including the following feature and steps that publishers need to complete:

1. Support multiple users with distinct permissions to administer the software and to manage the resources it hosts.
2. Upload source data as a delimited text file or connect to a database.
3. Map the terms (*e.g.*, fields or headers in a database or spreadsheet) from the source dataset to the terms in the Darwin Core standard (**Figure 2**).
4. Enter dataset metadata that specify scope, methodology, ownership, rights, etc.
5. Produce a Darwin Core Archive and a publicly accessible web page (**Figure 3**) that shows the metadata and links to the archive and other documents that were created.
6. Register datasets with the GBIF registry (http://www.gbif.org/dataset) so they are discoverable and can be harvested for indexing by GBIF and others.

### Simple Darwin Core and Extensions

The IPT supports the publication of two types or “cores” of Darwin Core Archives. The first and most common type is the Occurrence Core, which consists of occurrence records (*e.g.*, museum specimens or observations). The second type is the Taxon Core, which is used for checklists, and contains taxon records (*e.g.*, a record of the occurrence of a species, as opposed to the occurrence of an individual organism of that species in nature). Darwin Core Archives created for taxon checklists have the same advantages as archives for occurrence datasets – easy mobilization, aggregation, and interoperability.

The majority of occurrence and taxon data can be represented as Simple Darwin Core (http://rs.tdwg.org/dwc/terms/simple/): a flat file specification with rows and columns. Both the Occurrence Core (http://rs.gbif.org/core/dwc_occurrence.xml) and Taxon Core (http://rs.gbif.org/core/dwc_taxon.xml) are Simple Darwin Core representations with their own subset of Darwin Core terms.

However, the data are often richer than can be structured in a flat, Simple Darwin Core record, thus data publishers needed a way to represent that richness. The mechanism in the IPT for adding this richness to a Darwin Core Archive is known as an “extension”. GBIF maintains a registry of such extensions (http://rs.gbif.org/extension/) that can be used in the IPT. Extension records are meant to be related many-to-one to core records, constituting a star (http://www.dwhworld.com/dwh-schemas/) in Darwin Core Archives. In this way, data such as multiple images, measurements, or identifications, can be associated many-to-one with a single occurrence record. Information within a Darwin Core Archive, from linked extension data all the way up to the metadata for the entire dataset, provide a semantically rich aggregation that lends itself well to the notion of a reusable and linkable Research Object [22].

Prior to Darwin Core Archives and extensions, related information of this nature in Darwin Core (but not in other data standards such as ABCD [23]), relied on multiple entries concatenated into a single field. For example, Darwin Core offers the term *dwc:associatedMedia*, in which links to all media associated with a specimen can be added, with each link separated by a delimiter. Media objects themselves, however, are complex, having their own title, description, and rights. For the example of media, the Simple Multimedia Extension (http://rs.gbif.org/extension/gbif/1.0/multimedia.xml) now provides the means to share this richer information. In a Darwin Core Archive, this is done by relating a key identifying field in the extension to the unique id of a record in the core, whether an Occurrence Core or a Taxon Core.

Unlike the Simple Multimedia extension, which can be related to either an Occurrence Core or a Taxon Core, some extensions are specific to one core data type. The Germplasm extension (http://rs.gbif.org/extension/germplasm/20120911/), for example, is a means to relate Darwin Core Occurrence records with Multi-Crop Passport descriptors (http://www.bioversityinternational.org/uploads/tx_news/1526.pdf) through a distinct vocabulary maintained by the plant genetic resources community. For taxonomic checklists, the IPT provides a set of extensions, constituting the Global Names Architecture (GNA) profile (http://www.gbif.org/resources/2562), which links species to vernacular names, geographic distributions represented as ranges, type designations, and bibliographic references. Extensions allow a specialized subset of the broader community to expand upon the capabilities for biodiversity data sharing within their domain.

Endresen and Knüpffer [24] described the extension creation process. The main steps are to 1) create initial vocabularies and make terms available via the GBIF resource registry, 2) properly encode the terms in XML format so that they can be parsed by vocabulary management tools that GBIF maintains, and 3) load the extension into the GBIF resource registry. GBIF and the community of developers provide some oversight to assure that extensions provide useful services. The most difficult part of the creation of a new extension is in the management of vocabularies and the assurance that terms used within extensions are developed according to best practices, such as the Simple Knowledge Organization System (SKOS) framework (http://www.w3.org/2004/02/skos/) for describing and resolving concepts and terms.

## Results and Discussion

### Developing community training with the IPT

The IPT was built to help simplify data publishing steps for publishers. The previous publishing systems provided insufficient performance, were less scalable, and were hard to manage locally. The IPT simplifies these processes, but still requires some specialized skills and knowledge to properly go from local databases to published Darwin Core Archives. When the IPT was first released, the challenge was to get it adopted by the community. In order to develop a base of expert trainers, GBIF hosted two workshops to “train the trainers”, which GBIF calls a “distributed helpdesk system” (more information on the workshops here: http://www.gbif.org/resources/2696).

The success of efforts to develop a distributed helpdesk can be measured in part by adoption of the IPT (discussed below) and in part via anecdotal information from those early adopters and experts. From the lens of projects such as VertNet (http://vertnet.org) and Canadensys (http://www.canadensys.net/), which are utilizing the IPT for its network of data publishers, the training workshops paid immediate dividends. The initial workshops helped to train expert-level IPT administrators who could then further disseminate knowledge and skills across the respective networks through the development of step-by-step guides, such as the guide from Desmet and Sinou (http://www.canadensys.net/data-publication-guide) [25]. The rapid diffusion of knowledge has resulted in a more capable set of local IPT users empowered to do core publishing tasks.

The IPT further promotes community organization through expert-run IPT instances serving a community of users. Expert-operated instances are less prone to maintenance problems over time, and save resources (servers and technical expertise). A thematic example of this is VertNet, which supports publishers having their own IPT instances or allows them to use an VertNet-hosted instance (http://vertnet.nhm.ku.edu:8080/ipt/). Most publishers have chosen to use the VertNet IPT for its sheer convenience. Canadensys and other country-level nodes are effectively building data publishing networks based on single installations or constellations of IPTs, often with consistent branding.

### Growth of the Integrated Publishing Toolkit

**Figure 4:**
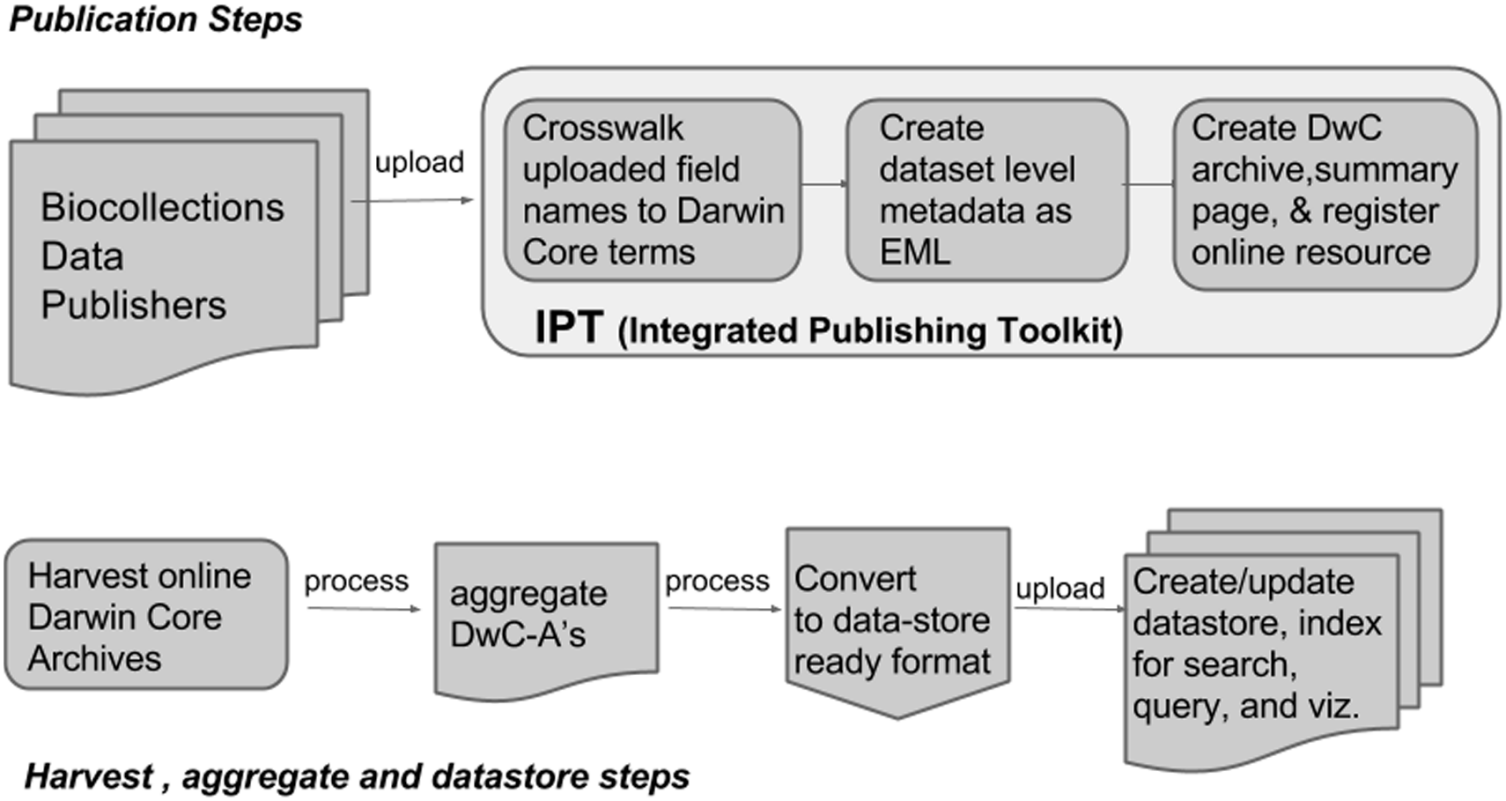
The current workflow for biodiversity data networks has multiple steps that separate the publishing of datasets from downstream aggregation and enhanced discoverability. The IPT supports the creation and publication of Darwin Core Archives accessible for download, with a publicly available summary web page. Aggregators harvest, process, and upload Darwin Core Archives into systems effective for searching, filtering, visualization, and download.

IPT has quickly become a widely used publication tool for GBIF data publishers. The impact of the transformation in the data publication workflow is readily seen in public statistics maintained by GBIF on IPT installations (http://www.gbif.org/ipt/stats). IPT, as of April 2014, is supporting publication of 220 million records coming from 872 occurrence datasets served through 128 installations. Although there are many factors that lead to a software implementation being successful, the IPT has clear advantages compared to previous publishing approaches. First, the IPT finally allowed a complete data mobilization workflow, from in-house data management systems to GBIF, all standardized and discoverable. Second, the IPT fills the role of a computer-aided guide, lowering the technical threshold for data publishing. Although IPT installations require some technical skill and understanding of best practices and data standards, they are practical to install and manage by local technical staff. Alternatively, many organizations (*e.g.*, VertNet and Canadensys) provide technical hosting of an IPT for institutions who opt not to host an IPT of their own. These hosted IPTs can support a multitude of institutions in a single installation. Third, the IPT decouples publishing steps from downstream operations such as harvesting, aggregating, and developing new access points to the data (**Figure 4**). Finally, offering data in bulk and in a machine-readable format follows open data publication best practices (http://opendatahandbook.org/en/how-to-open-up-data/make-data-available.html).

### Newest versions of the IPT

Since the release of version 2.0, the IPT has been customizable and available in multiple languages, currently including Portuguese, French, Spanish, Traditional Chinese, Japanese, and English. Customization provides the means to enhance the user interfaces so that IPT instances can be branded by institutions that maintain the software and provide direct access to resources. In the latest version of the IPT, customization has been further simplified via a custom CSS file that overrides default look and feel. Excellent examples of such a customized IPT installations are Canadensys (http://data.canadensys.net/ipt/) and Sistema de información sobre biodiversidad de Colombia (http://ipt.sibcolombia.net/sib/).

The current version of the Integrated Publishing Toolkit (2.1.1, as of April 2014 also includes a number of improvements over earlier versions. For example, past versions of the IPT did not include dataset versioning. One could republish a Darwin Core Archive, but each publishing event erased the previous one. In IPT v2.1.1, versioning is supported for dataset metadata and optional for data (more details here: https://code.google.com/p/gbif-providertoolkit/wiki/IPT2ManualNotes?tm=6#Published_Release). In addition, if a dataset was previously published through one of the former publishing methods (DiGIR, TAPIR, etc.), the same GBIF registry entry can be maintained (though updated) to reflect the IPT as the new publishing method. Also new in the latest version of the IPT, a resource can be configured to publish automatically on a schedule (*e.g.*, annually, bi-annually, monthly, weekly, or daily). When automated publishing is enabled, the publishing interval and next published date are clearly displayed. Finally, IPT v2.1.1 assures that each published record has a unique occurrence identifier. If missing or duplicate records are found during publishing, they are flagged and the publishing process halted. Transitioning to widespread use of stable occurrence identifiers greatly simplifies tracking individual records both within the GBIF network and as records propagate out of the network.

The latest versions of the Integrated Publishing Toolkit also include a number of significant improvements over earlier versions. Since IPT v2.0.5, versioning is supported for dataset metadata and optional for data (more details here: https://code.google.com/p/gbif-providertoolkit/wiki/IPT2ManualNotes?tm=6#Published_Release). This allows publishers to republish a Darwin Core Archive without erasing previous versions and allows users to reference a specific version of a dataset. Also new in that version of the IPT is that a resource can be configured to publish automatically on a schedule (*e.g.*, annually, bi-annually, monthly, weekly, or daily). When automated publishing is enabled, the publishing interval and next published date are clearly displayed. In addition, if a dataset was previously published through one of the former publishing methods (DiGIR, TAPIR, etc.), the same GBIF registry entry can be maintained (though updated) to reflect the IPT as the new publishing method. The current version of the IPT (2.1.1, as of April 2014) introduces data validation of record IDs (occurrenceID or taxonID). If mapped by the user, the IPT now assures that each published record has a unique identifier. If missing or duplicate record IDs are found during publishing, they are flagged and the publishing process halted. Transitioning to widespread use of stable occurrence and taxon identifiers greatly simplifies tracking individual records both within the GBIF network and as records propagate out of the network, and allows additional features to be build upon these.

### The IPT and traditional scientific publishing

The IPT has not only supported open data publishing via the GBIF network, but can also act as a repository for occurrence or checklist data referenced in a paper. One such example is the published description of a new species of leafcutter bee, *Megachile (Megachiloides) chomskyi* [24] in the journal Zookeys. The paper makes explicit reference to the new records provisioned via the IPT (http://data.canadensys.net/ipt/resource.do?r=megachile_chomskyi), including a digital object identifier (DOI) allowing the reader to simply click the link (http://doi.org/10.5886/txsd3at3) and retrieve the data. The main advantage over general data repositories, such as Dryad (http://datadryad.org) and Figshare (http://figshare.com), is that the IPT enforces the data to be standardized as a Darwin Core Archive, which increases usability.

Newer versions of the IPT (after version 2.0.2) help authors move beyond archiving for publication, and into directly creating publications, such as “data papers” [27]–[28]. Data papers are scholarly publications that simultaneously describe and provide access to datasets, providing a means for a dataset to be cited in the same manner as other literature contributions. Pensoft Publishers (http://www.pensoft.net) is a pioneer in this domain, and recently launched a new journal devoted to data publishing: The Biodiversity Data Journal (http://biodiversitydatajournal.com/). Chavan and Penev [27] discuss the process of generating data papers in more detail, but the main feature the IPT provides is the means to export the dataset metadata into a rich text format that has most of the needed sections for a data paper manuscript. This manuscript can then be submitted for peer review to the journal publisher. One example of such a data paper is Desmet and Brouillet [29], which describes a national checklist of vascular plants.

## Conclusions

The Integrated Publishing Toolkit continues to evolve. Key forthcoming improvements include 1) expanding to new types of input data sources and, 2) simplifying installations and upgrades, which often require assistance from the GBIF Helpdesk. More long-term improvements include: 1) providing the means to associate a Digital Object Identifier (DOI) with each dataset during a publication event in order to facilitate tracking of usage and impact; 2) setting up the means to annotate records from Darwin Core Archives and have them populated back to the source archive; 3) creating tools to validate and clean data within Darwin Core Archives during the IPT publishing process; 4) opening the IPT to more collaborative development; and 5) ensuring that IPTs participate in networks that provide data redundancy – secondary copies of data will be stored for disaster recovery purposes.

Data publication is a growing domain in the life sciences [28], and one key area for further growth using the IPT will be to provide metrics of data use to the original publishers. This is a difficult, multi-faceted endeavor with many possible solutions. Part of the way forward may be to associate a Digital Object Identifier (DOI) with the IPT summary page. The IPT already allows resolvability of the dataset via a URL to the summary page, but adding a DOI to this summary page would provide a resolvability mechanism using services well established in the publishing industry [30]. In addition, data consumers could make annotations about individual data records if they were resolvable. Such a mechanism, long discussed in biodiversity informatics (Filtered Push, http://wiki.filteredpush.org/wiki/), would link downstream assertions directly to the original records, so that publishers as well as the rest of the community could track possible data improvements. Prototyping DOI assignment is already happening. Published datasets in Canadensys, for example, now have DOIs issued by DataCite Canada.

Ad-hoc annotations are only one mechanism to improve data quality and fitness for use. Records from Darwin Core Archives are well understood semantically and syntactically. Darwin Core Archives do not represent the data semantically in RDF, but it is an area of on-going research to create tools to translate data from Darwin Core Archives into RDF for use in semantic frameworks such as Linked Open Data (https://code.google.com/p/tdwg-rdf/wiki/DwcRdfGuideProposal). They can be easily ingested, processed, and rewritten, providing a means for immediate post-publication data improvements. A future complement to the IPT will be to build data quality tools that can be leveraged at multiple points in the publishing process. Adding data quality tools to the IPT remains a challenge. Network-wide cleaning approaches have been attempted in the past [17] with limited success. For smaller datasets, the IPT could be linked to tools that run simple checks for outliers, non-standard values, including taxon name issues. For larger datasets (*e.g.*, eBird with over 150 million data records and growing), local cleaning processes linked to the IPT may be impractical. Another option would be to provide a set of pluggable remote validation services that can access either Darwin Core Archives or that might be usable within the GBIF portal (or other portals). Those tools could report back to publishers and allow for republication via the IPT once possible errors are checked and corrected.

The Integrated Publishing Toolkit has become a lynchpin piece of software, in a fast-growing distributed biodiversity network architecture, connecting publishers into the system and supporting essential functions such as updating and archiving previous dataset versions. Sequential improvements, not just to the IPT, but across this architecture, continue to lead to a more robust, scalable and sustainable future for what is surely the largest globally distributed, consistently formatted and structured, biodiversity data sharing initiative ever built.
